# No Increased Risk of Infection Following a Protocol Change to Decrease Duration of Perioperative Antibiotic Prophylaxis in Liver Transplantation

**DOI:** 10.1111/tid.70192

**Published:** 2026-02-25

**Authors:** Hutton Brandon, Crystal M. Truax, Eduardo Rodriguez Zarate, Brandon J. Tritle, Hannah Imlay

**Affiliations:** ^1^ Division of Infectious Diseases Department of Internal Medicine University of Utah Salt Lake City Utah USA; ^2^ Department of Pharmacy University of Utah Health Salt Lake City Utah USA; ^3^ Transplant Hepatology Tampa General Medicine Group Tampa Florida USA

**Keywords:** antibiotic prophylaxis, antimicrobial stewardship, liver transplant, perioperative prophylaxis

## Abstract

**Background:**

Liver transplant recipients are at high risk of bacterial surgical site infection (SSI) following transplantation. However, they also have increased risks of antibiotic complications such as *C. difficile* and resistant organism infections. The optimal duration of perioperative antibiotic prophylaxis for liver transplantation is uncertain.

**Methods:**

We analyzed infection outcomes in a total of 192 patients before and after a protocol change that shortened antibiotic prophylaxis duration at our institution. Durations were risk‐stratified with longer durations for patients hospitalized prior to liver transplantation. The recommended duration of antibiotic prophylaxis changed from 5 days or until transfer out of intensive care to 3 days for patients hospitalized prior to transplant and from 2 days to 1 day for all other patients.

**Results:**

There was no increased rate of SSI or bacteremia following the decrease in duration, with a change from 2.4% to 2.8% in patients hospitalized prior to transplant and 7.5% to 2.9% in other patients. Risk of SSI or bacteremia was not significantly increased in a multivariable model (OR 0.50, 95% CI 0.07–2.24; *p* = 0.41). There was no significant difference in readmission, graft rejection, graft failure, or mortality. Rates of acute kidney injury, *C. difficile* infection, and development of resistant organisms were not significantly different.

**Conclusion:**

Shortening the duration of perioperative antibiotic prophylaxis in liver transplantation does not increase the risk of infection and may reduce harms of antibiotic exposure.

## Graphical abstract

There was no increased rate of surgical site infection or bacteremia in liver transplant recipients following a decrease in perioperative antibiotic prophylaxis duration, with a change from 2.4% to 2.8% in patients hospitalized prior to transplant and 7.5% to 2.9% in other patients.

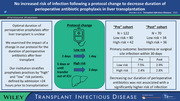

**Abbreviations**
AKI: acute kidney injury
AST: American Society of Transplantation Infectious Diseases Community of Practice
DCD: donor after cardiac death
IV: intravenous
LOS: length of stay
LT: liver transplant
LTRs: liver transplant recipients
MELD: Model for End‐Stage Liver Disease
OPTN: Organ Procurement and Transplantation Network
SRTR: Scientific Registry of Transplant Recipients
SSI: surgical site infection

## 1 Introduction

Liver transplant recipients (LTRs) face a high risk for bacterial infection in the first 30 days following transplant [1], in large part due to surgical site infections (SSIs) that occur in 7%–30% of LTRs [2, 3, 4]. Perioperative prophylaxis is administered at the time of transplant surgery to reduce the risk of bacterial SSI, but there are few data that inform perioperative antibiotic duration specifically for liver transplantation [5, 6]. LTRs are also at high risk for antibiotic‐related complications, such as *C. difficile* infection (incidence of 3.5%–12.4%) [7] and development of resistant organisms (21.7%–25% in the first year post‐transplant) [8], so optimizing antibiotic duration and choice, including antimicrobial prophylaxis strategies, is a priority for antimicrobial stewardship programs [9]. Risks of complications from antibiotics and resistance must be weighed against risks of SSI.

The American Society of Transplantation Infectious Diseases Community of Practice (AST) provided perioperative antibiotic prophylaxis recommendations specifically for liver transplant in 2019 [6]; these included a third‐generation cephalosporin with ampicillin, piperacillin‐tazobactam alone, or ampicillin‐sulbactam alone for ≤ 48 h. This recommendation cited only one study on duration in 26 patients comparing 2–3 days prophylaxis to 4–7 days [10]. Although several risk factors for SSI are known, including high pre‐transplant MELD, the AST recommendations for antibiotic duration or choice did not differ based on the presence of SSI risk factors.

Since the 2019 AST guideline was published, two studies have attempted to determine the optimal duration for perioperative antibiotic prophylaxis. One study was a randomized controlled trial of 102 LTRs compared 72 h of perioperative antibiotic prophylaxis to only intra‐operative dosing and found no significant difference in incidence of SSI [2]. However, the study excluded patients who were admitted prior to transplantation, which may have differentially excluded patients at high‐risk for worse clinical outcomes (e.g., those with high MELD or acute liver failure). The second study was a retrospective observational study of 68 LTRs, including those admitted prior to transplantation. This study demonstrated no significant difference in incidence of SSI between 72‐ and 24‐h durations of perioperative antibiotics [3].

Following a protocol change to reduce the duration of perioperative antibiotic prophylaxis in our institutional liver transplant guideline, we examined the impact of shorter durations of perioperative prophylaxis in both patients admitted at the time of liver transplantation and those admitted prior to transplant.

## 2 Methods

### 2.1 Study Design

We performed a retrospective cohort study to evaluate outcomes before and after a protocol change in our perioperative antibiotic prophylaxis protocol for patients ≥ 18 years old undergoing liver transplant (LT) at the University of Utah from January 1, 2018 to June 17, 2022.

### 2.2 Institutional Prophylaxis Practices

Our institutional prophylaxis durations are stratified by the duration of time patients are admitted prior to transplant—as a proxy for whether they experienced medical complications or liver failure requiring admission prior to transplant. Patients admitted < 24 h prior to transplant have been traditionally suspected to be “low‐risk” for complications (Group A) compared to those admitted ≥ 24 h prior to transplant (Group B) who have been traditionally suspected to be “high‐risk” for complications as they typically had more fulminant liver failure, were more ill, and thus perceived to be at higher risk for infection. Patients who have been admitted for < 24 h prior to transplant have received less intense preventive strategies relative to the “high‐risk” group. As part of a regularly timed update of institutional guidelines, the perioperative antibiotic prophylaxis protocol changed to recommend a reduced duration for both risk groups on January 19, 2021, after a multidisciplinary meeting of liver transplant surgeons, transplant pharmacists, and infectious diseases physicians. Although shortening prophylaxis durations to 24 h in both Group A and Group B in accordance with guidelines was discussed [6, 11], a longer duration of perioperative prophylaxis was preferred for Group B because “high‐risk” patients had not been sufficiently studied and were perceived to be at increased risk of infection and mortality. In addition, although contrary to AST recommendations, vancomycin was given as part of the perioperative regimen due to the suspicion that this group may have a higher rate of resistant organisms causing post‐operative infection. As a result, our “pre” and “post” prophylaxis regimens were as follows:

“Pre” protocol:

Group A: intravenous (IV) piperacillin‐tazobactam initiated intra‐operatively and given for 48 h following LT (regimens are individualized for patients with penicillin allergy or history of specific resistant infections).

Group B: IV vancomycin and piperacillin‐tazobactam initiated intra‐operatively and continued for 5 days or until transfer out of ICU, whichever was longer.

“Post” protocol:

Group A: IV piperacillin‐tazobactam intra‐operatively and for 24 h after LT.

Group B: IV vancomycin and piperacillin‐tazobactam intra‐operatively and for 72 h after LT.

We considered January 1, 2018 to January 19, 2021, as the “pre” period, and January 20, 2021 to June 17, 2022, as the “post” period.

### 2.3 Study Subjects

We included consecutive patients who underwent LT during our study period and excluded patients who died during transplant surgery. If a patient received a second liver transplant during the same hospitalization, only the first transplant event was included in our analysis, and follow‐up time was censored at the time of the second transplant. Per institutional protocol, most study subjects received corticosteroids for standard induction immunosuppression. Maintenance immunosuppression consisted of tacrolimus, mycophenolic acid, and prednisone.

### 2.4 Study Outcomes

We pursued two primary study analyses. First, we examined adherence to our institutional prophylaxis guidelines’ recommended antibiotic choice and duration in the pre‐ and post‐time frames among Group A and Group B.

Second, we examined clinical outcomes associated with the protocol change. Our primary clinical outcome was a composite of bacteremia or microbiologically‐defined SSI within 30 days of LT. Bacteremia was defined as the presence of an organism identified from blood cultures within 30 days of transplant, excluding coagulase‐negative staphylococci that grew from only one of two sets of blood cultures. SSI was defined as a positive culture from surgical or percutaneous sampling performed for suspected infection within 30 days of transplant.

Secondary outcomes included acute kidney injury (AKI), infection with a resistant organism, *C. difficile* infection, length of stay (LOS), mortality at 30 days, and readmission at 30 days, graft rejection, and graft failure at 100 days. AKI was defined as an increase in serum creatinine of ≥ 0.3 mg/dL in 48 h or an increase to 1.5 ×  higher than baseline within a 7 day period, or new or continued need for renal replacement therapy, as defined by the Kidney Disease Improving Global Outcomes (KDIGO) definitions of AKI [12]. Infection with a resistant organism was defined as the presence of MRSA, VRE, Enterobacterales with phenotypic ceftriaxone resistance, *Pseudomonas* with resistance to ≥ 3 drug classes to which it is typically susceptible, *Acinetobacter*, or *Stenotrophomonas*. Since some cases of suspected SSI may not have had associated bacteremia, surgical sampling, or percutaneous sampling to yield culture results, we performed a sensitivity analysis using a separate composite outcome that included a change in antibiotic therapy within 30 days post‐transplant for suspected infection in addition to our primary outcome. Similarly, we assessed the risk of bacteremia and SSI within 100 days post‐transplant.

We also performed a separate analysis stratifying by short (≤ 72 h) versus long (> 72 h) duration of perioperative antibiotics to assess differences between these groups following the intervention. Additionally, we performed an analysis to assess possible risk factors to explain the increased risk of SSI we found associated with recipients of donor after cardiac death (DCD) livers and an analysis to assess the risk of AKI associated with vancomycin exposure in Group A (Group B was not analyzed due to near universal vancomycin use).

### 2.5 Data Collection

Baseline demographic and clinical data were obtained from a prospectively‐maintained database of consecutive LTs performed at our center, along with demographic and clinical characteristics. Additional demographic and clinical data were obtained from the University of Utah Enterprise Data Warehouse. The remainder of the data including perioperative antibiotic choice and duration were obtained from manual chart extraction. This study was approved by the University of Utah Institutional Review Board.

### 2.6 Data Analysis

Chi‐squared and Fisher's exact test were used to analyze dichotomous variables, and the Wilcoxon rank sum test was used to analyze continuous variables; a *p* value of < 0.05 was considered statistically significant. Univariable and multivariable logistic models were used to assess risk factors for key clinical outcomes. Because a small number of patients developed the primary endpoint, we limited the number of covariates that we included in our multivariable models [13]. In a multivariable model, we examined factors that we considered potential confounders or associated with our composite definition of infection, including presence of diabetes, DCD, MELD > 35, or body mass index > 30. We forced the risk group (high‐ vs. low‐risk) and pre‐/post‐cohort into our model and manually implemented backward elimination to identify the most important remaining variable in the model. Using this strategy, we chose patient cohort (pre‐ vs. post‐protocol change), risk group (high vs. low‐risk), and DCD as covariates in our multivariable models of composite outcomes to derive our adjusted analysis. All data analyses were performed in R [14, 15, 16].

## 3 Results

### 3.1 Study Population

From January 12, 2018 to June 17, 2022, 196 LT procedures were performed at our center for 193 patients. Follow‐up time for three patients was censored at the time of their second liver transplant. We excluded one LTR who died intra‐operatively prior to completing planned antibiotic prophylaxis. Therefore, our final cohort was 192 patients. The pre‐intervention cohort consisted of 122 patients (80 in Group A and 42 in Group B), and 70 patients (34 in Group A and 36 in Group B) were included in the post‐intervention cohort.

The pre‐intervention cohort had a higher proportion of patients of age ≥ 65 years (24% vs. 10%) and a lower proportion of patients in Group B (34% vs. 51%). Cohorts were otherwise similar (Table 1).

**TABLE 1:** Demographics.

	Overall, *N* = 192 (%)	Pre‐protocol change, *N* = 122 (%)	Post‐protocol change, *N* = 70 (%)
Age group (years)			
≥ 65	36 (19)	29 (24)	7 (10)
18–44	44 (23)	24 (20)	20 (29)
45–64	112 (58)	69 (57)	43 (61)
Male, *n* (%)	123 (64)	81 (66)	42 (60)
Patient defined race			
American Indian and Alaska Native	7 (3.6)	3 (2.5)	4 (5.7)
Asian	2 (1.0)	2 (1.6)	0
Native Hawaiian and Other Pacific Islander	1 (0.5)	0	1 (1.4)
White or Caucasian	161 (84)	102 (84)	59 (84)
Other	21 (11)	15 (12)	6 (8.6)
Ethnicity			
Hispanic/Latino	28 (15)	20 (16)	8 (11)
Not Hispanic/Latino	162 (84)	102 (84)	60 (86)
Chose not to disclose	2 (1.0)	0	2 (2.9)
Previous transplant	2 (1.0)	2 (1.6)	0
MELD score, median (Q1, Q3)	27 (21, 35)	25 (20, 33)	31 (23, 38)
Listed 1A	5 (2.6)	3 (2.5)	2 (2.9)
Surgery duration, minutes (Q1, Q3)	346 (294, 406)	350 (305, 411)	336 (277, 385)
Diabetes	85 (44)	55 (45)	30 (43)
Presence of beta‐lactam allergy	27 (14)	15 (12)	12 (17)
Admitted ≥ 24 h prior to transplant	78 (41)	42 (34)	36 (51)
Cold ischemia time, minutes, median (Q1, Q3)	338 (272, 442)	341 (277, 463)	319 (263, 403)
Antibiotic exposure within 72 h prior to transplant^a^	92 (48)	51 (42)	41 (59)
SBP prophylaxis prior to transplant	22 (11)	13 (11)	9 (13)
Steroid exposure prior to transplant	4 (2.1)	1 (0.8)	3 (4.3)
Other immunosuppressants prior to transplant^b^	16 (8.3)	10 (8.2)	6 (8.6)
Donor type			
Living	31 (16)	23 (19)	8 (11)
DBD	153 (80)	94 (77)	59 (84)
DCD	8 (4.2)	5 (4.1)	3 (4.3)
CMV serostatus			
D−/R−	33 (17)	21 (17)	12 (17)
D−/R+	44 (23)	26 (21)	18 (26)
D+/R−	41 (21)	23 (19)	18 (26)
D+/R+	74 (39)	52 (43)	22 (31)

Abbreviations: D, donor CMV IgG; DBD, donation after brain death; DCD, donation after cardiac death; MELD, Model for End‐Stage Liver Disease; Q1, first quartile; Q3, third quartile; R, recipient CMV IgG; SBP, spontaneous bacterial peritonitis.

^a^ Some patients received multiple antibiotics: amoxicillin‐clavulanate = 4, cefepime = 2, cefpodoxime = 4, ceftriaxone = 8, ciprofloxacin = 27, clindamycin = 1, daptomycin = 2, doxycycline = 2, levofloxacin = 4, meropenem = 6, metronidazole = 2, piperacillin‐tazobactam = 35, trimethoprim‐sulfamethoxazole = 3, vancomycin = 25.

^b^ Azathioprine = 4, golimumab = 1, hydroxychloroquine = 3, infliximab = 1, mycophenolate = 4, tacrolimus = 5, vedolizumab = 4.

Compared to Group A, Group B contained a higher percentage of patients who identified as Hispanic or Latino, had diabetes, or were exposed to antibiotics prior to transplant (Table S1). Group B had a higher median MELD score (36 vs. 23).

### 3.2 Antibiotic Use

**FIGURE 1:**
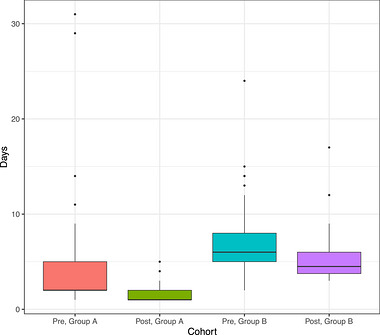
Antibiotic duration by cohort and risk group.

There was a significant decrease in median antibiotic duration in both Group A and Group B following the protocol change (Figure 1). The Group A median duration decreased from 2 (IQR 2–5) to 1 (IQR 1–2) days (*p* < 0.001) and the Group B median duration decreased from 6 (IQR 5–8) to 4.5 (IQR 3.8–6) days (*p* = 0.002) with fewer patients who received prophylaxis durations > 7 days (Group A: 10 (13%) to 0, *p* = 0.032; Group B: 16 (38%) to 4 (11%), *p* = 0.007) post‐intervention. Piperacillin‐tazobactam and vancomycin were the most used antibiotics, consistent with our institutional guidelines. Vancomycin use decreased in Group A following the protocol change (Figure S1).

### 3.3 Outcomes

### 3.4 Clinical Outcomes

Outcomes and complications by pre‐ and post‐cohort are presented in Table 2. Rates of our primary composite endpoint (bacteremia or microbiologically‐defined SSI) were similar after stratifying for risk status (*p *> 0.05 for all comparisons). Isolated organisms are enumerated in Table S2. Univariable analysis of the primary outcome of bacteremia or microbiologically‐defined SSI did not identify any significant difference associated with the pre‐ or post‐intervention cohorts (Table 3). In our multivariable model, there was no significant difference in bacteremia or SSI due to the protocol change after adjustment for high/low‐risk status and DCD donor (OR 0.5, 95% CI 0.07–2.24; *p* = 0.41). There was no significant difference in rates of bacteremia or SSI in patients who received ≤ 72 h or > 72 h duration of perioperative antibiotic prophylaxis stratified by risk group (Table S3).

**TABLE 2:** Outcomes and complications by risk group and protocol cohort

	Group A^a^		Group B^a^	
	**Pre‐protocol change, *N* = 80 (%)**	**Post‐protocol change, *N* = 34 (%)**	**Pre‐protocol change, *N* = 42 (%)**	**Post‐protocol change, *N* = 36 (%)**
Bacteremia or SSI within 30 days	6 (7.5)	1 (2.9)	1 (2.4)	1 (2.8)
Bacteremia within 30 days	3 (3.8)	0	0	0
SSI within 30 days	4 (5.0)	1 (2.9)	1 (2.4)	1 (2.8)
Bacteremia or SSI within 100 days	10 (13)	2 (5.9)	4 (9.5)	2 (5.6)
Infection composite^b^	31 (39)	14 (41)	19 (45)	17 (47)
Antibiotic change for suspected infection	31 (39)	14 (41)	19 (45)	17 (47)
Length of stay, days, median (Q1, Q3)	9 (7, 14)	8 (6, 11)	20 (10, 26)	14 (11, 25)
Readmission	26 (33)	9 (26)	7 (17)	6 (17)
30‐day mortality	0	0	0	0
AKI^c^	59 (74)	19 (56)	37 (88)	34 (94)
Creatinine increase >0.3 mg/dL at 48 h	51 (64)	17 (50)	23 (55)	22 (61)
Creatinine increase ≥1.5 × baseline at 7 days	31 (39)	6 (18)	18 (43)	10 (28)
Creatinine increase ≥1.5 × baseline at 14 days	8 (10)	1 (2.9)	5 (12)	5 (14)
Received renal replacement therapy	12 (15)	1 (2.9)	24 (57)	22 (61)
Resistant organism within 100 days	12 (15)	2 (5.9)	9 (21)	6 (17)
*Clostridioides difficile* infection within 100 days	9 (11)	2 (5.9)	4 (9.5)	3 (8.3)

*Note*: Methicillin‐resistant *Staphylococcus aureus* (*n* = 0), vancomycin‐resistant *Enterococcus* spp. (*n* = 12), Enterobacterales with ceftriaxone resistance (*n* = 21), *Pseudomonas* with resistance to ≥ 3 drug classes to which it is typically susceptible (*n* = 2), *Acinetobacter* spp. (*n* = 0), or *Stenotrophomonas* spp. (*n* = 0).

Abbreviations: AKI, acute kidney injury; Q1, first quartile; Q3, third quartile; SSI, surgical site infection.

^a^ Group A = hospitalized < 24 h prior to transplant surgery; Group B = hospitalized ≥ 24 h prior to transplant surgery.

^b^ Bacteremia, SSI, or antibiotic change for suspected infection within 30 days post‐transplant.

^c^ Defined by creatinine increase of 0.3 mg/dL in 48 h, an increase of 1.5 × in 7 days, or need for continued renal replacement therapy in the 14‐day post‐operative period.

**TABLE 3:** Univariable and multivariable models of primary outcome.

Bacteremia or SSI				
	**Univariable** **OR (95% CI)**	***p* value**	**Multivariable** **OR (95% CI)**	***p* value**
Intervention cohort	0.48 (0.07, 2.07)^a^	0.37	0.5 (0.07, 2.24)	0.41
Group B	0.40 (0.06, 1.72)	0.26	0.59 (0.08, 2.87)	0.54
Donation after cardiac death	8.43 (1.1, 45.3)	0.018	7.22 (0.9, 44.1)	0.037
Age ≥ 65 years	2.27 (0.46, 9.09)	0.26		
BMI ≥ 30	3.30 (0.84, 16.1)	0.10		
Diabetes	1.61 (0.41, 6.69)	0.49		
MELD ≥ 35	0.29 (0.02, 1.64)	0.25		

^a^ An OR < 1 is consistent with a lower risk of that outcome in the post‐intervention cohort than the pre‐intervention group cohort

### 3.5 Sensitivity Analysis

Our sensitivity analysis demonstrated that the proportion of patients who required a change in antibiotics due to suspected infection or had bacteremia or SSI was similar between the pre‐ and post‐intervention cohorts (Table 2). The protocol change was not associated with a significant change in risk of our composite definition of infection in both adjusted and unadjusted analyses (Table S4) or a significant change in risk of bacteremia or SSI within 100 days post‐transplant (Table S5).

### 3.6 Secondary Outcomes

There were no statistically significant differences between the proportion of patients who developed AKI, *C. difficile* infection, or infection with a resistant organism in pre‐ versus post‐intervention groups stratified by risk group (*p* values > 0.05 for all comparisons) (Figure S2). We observed a high overall rate of AKI of 78% in our sample, with 59% meeting our definition at 48 h post‐operatively, but only 34% meeting criteria at 7 days and 31% requiring renal replacement therapy. LOS was shorter in the post‐intervention cohort in Group A (median 8 vs. 9 days; *p* = 0.040) but not in Group B (median 14 vs. 20 days; *p* = 0.60) (Table 2). The rate of readmission stratified by risk group was not significantly different between intervention cohorts. There was no significant difference in rates of graft rejection (Group A 5% vs. 8.8%, *p* = 0.42; Group B 4.8% vs. 8.3%, *p* = 0.66) or failure (Group A 2.5% vs. 0; Group B 0 vs. 0) within 100 days of transplant. There were no deaths within 30 days of transplant in our studied patients. Stratifying by whether patients received ≤ 72 h of perioperative antibiotic prophylaxis, there were no statistically significant differences in secondary outcomes between antibiotic duration groups except LOS, which was shorter in the cohort that received ≤ 72 h of perioperative antibiotic prophylaxis (Table S3). Relative to Group A, Group B did not have a significantly higher rate of post‐operative bacteremia or microbiologically defined SSI (2.6% vs. 6.1%; *p* = 0.32). Incidence of bacteremia or SSI was significantly higher in DCD recipients than other LTRs (25% vs. 3.8%, *p* = 0.047); however, there were only 8 DCD recipients (Table S6). Vancomycin exposure was not associated with a significantly higher risk of AKI in Group A (OR 0.97, 95% CI 0.44–2.19, *p* = 0.95).

## 4 Discussion

Post‐operative infection is a significant complication of liver transplantation. In this study, 4.7% of patients developed microbiologically‐defined SSI or bacteremia within 30 days following transplantation despite perioperative antibiotic prophylaxis. Decreasing the recommended duration of perioperative antibiotic prophylaxis from 48 to 24 h in patients admitted at the time of transplantation and from 5 days or duration of ICU stay to 72 h in patients admitted prior to transplantation was not associated with a significantly higher incidence of SSI or bacteremia in univariable or multivariable analyses. We also did not observe an increased risk of SSI associated with higher MELD or admission prior to transplantation. These results are consistent with prior studies and suggest that shorter durations of perioperative antibiotic prophylaxis during liver transplantation are equally effective at preventing post‐operative infection. Additionally, stratifying prophylaxis by duration of admission prior to transplant may not be beneficial.

Our findings are consistent with prior laboratory and clinical studies that have not shown increased risk of SSI with shorter durations of perioperative antibiotic prophylaxis [2, 3, 10]. Perioperative prophylaxis is thought to prevent SSI by preventing pathogenic bacteria, introduced from incisions of non‐sterile sites such as the skin or enteric tract, from establishing infection when they contaminate the surgical bed, and thus the initial dose of antibiotics provides the benefit [17]. In animal models, antibiotic dosing prior to the incision is more effective than antibiotics given after the time of incision closure; extending antibiotics beyond surgery does not affect the rate of infection [18, 19, 20]. In humans, prolonged antibiotic prophylaxis has not improved SSI prevention for most types of surgery [17, 21]. Liver transplantation is a prolonged surgery with the potential for contamination introduced from biliary and enteric sites as well as immunosuppression impairing host immune response, so it is plausible that prolonged prophylaxis could be beneficial [22]. There are no large randomized controlled trials that have examined short versus long antibiotic prophylaxis at liver transplant among patients with and without fulminant liver failure. However, randomized controlled trials of biliary procedures and renal transplantation have not demonstrated a significant benefit from prolonging prophylaxis [23, 24]. Our findings, and those of the three prior studies on prophylactic perioperative antibiotic duration in LTRs, also do not show that shorter prophylaxis increased risk of SSI or perioperative infection [2, 3, 10].

Our center stratifies prophylactic antibiotic choice and duration by whether patients are hospitalized prior to transplantation as a proxy for those who may be more unstable or have acute liver failure. We did not observe an increased risk of SSI or other infection associated with higher MELD or admission prior to transplantation. Group B did not have increased rates of infection or mortality compared to Group A, despite having a higher median pre‐transplant MELD score, although post‐operative LOS was higher (median 9 vs. 15 days). Perioperative antibiotic duration was longer and the spectrum of coverage broader in Group B than in Group A, which could explain the lack of increased rates of infection. However, the available prior data do not suggest that longer post‐operative antibiotic exposure or increased vancomycin exposure would decrease rates of infection, and this finding is consistent with several other studies evaluating risk factors for SSI in LTRs [3, 4]. Although higher MELD is associated with decreased graft and patient survival, it is not consistently associated with a higher risk of SSI [2, 4, 22, 25, 26, 27, 28, 29]. Our findings support consensus recommendations for perioperative prophylaxis in liver transplant that do not recommend a different antibiotic choice or duration based on pre‐transplant hospitalization or higher MELD scores [6]. Furthermore, a recent survey of transplant center practices indicated that a minority of centers (30%) stratify antibiotic choice or duration into “high” or “low‐risk” candidates [30].

In our cohort, we also did not observe an increased risk of antibiotic‐associated complications in patients receiving a longer duration of antibiotics. However, the risks of prolonged antibiotic exposure have been clearly demonstrated in other populations. LTRs are known to have higher rates of infection with *C. difficile* infection and resistant organisms [7, 8]. A larger study has shown a higher risk of *C. difficile* infection and AKI with perioperative prophylaxis extended beyond 48 h [21]. More generally, extended durations of antibiotics have been linked to increased risk of complications and infection with resistant organisms [31, 32]. Our study was likely underpowered to detect these differences. We observed a higher overall rate of AKI than has been previously noted in LTRs, likely because we used a sensitive definition of AKI (Stage 1 AKI per the KDIGO guidelines) [12, 33]. Antimicrobial stewardship initiatives that limit unnecessary antibiotic exposure are suggested in organ transplantation and other populations [9]. Optimizing perioperative antimicrobial prophylaxis is an opportunity for stewardship in organ transplantation [34].

Notably, use of a DCD donor was significantly associated with bacteremia or SSI in our multivariable analysis. These grafts can have a longer donor warm ischemia time, leading to impaired graft function, increased risk of biliary anastomotic failure, and increased rates of hepatic artery thrombosis [35]. Graft survival is lower in DCD per OPTN/SRTR data [25]. Of note, post‐transplantation outcomes using DCD organs, including graft function and survival, have improved after the introduction of perfusion machines [36].

This study had several limitations. While we used the protocol change to assess changes in perioperative prophylaxis duration, the retrospective nature makes it difficult to determine the impact of unmeasured confounders and changes in outcomes with time. Gathering data retrospectively also made it difficult to ascertain the precise duration for which the antimicrobials were intended for the purpose of prophylaxis. Our study was underpowered to measure small differences in our primary outcome and assessment of complications of antibiotic use but represents a real‐world cohort. Risk‐stratifying by duration of admission prior to liver transplantation may decrease the generalizability of our findings, as many other centers may not risk‐stratify perioperative antibiotic choice or duration. Similarly, the use of vancomycin and the antibiotic durations in Group B differ from consensus guidelines, which may limit generalizability. However, a recent survey of perioperative antimicrobial prophylaxis practices at 36 US transplant centers presented at IDWeek 2025 demonstrated substantial variation across centers, including: use of vancomycin in a minority (8%) of centers; durations of ≥ 72 h in at least some patients in 19% of centers, and the practice of risk‐stratification of antibacterial coverage/duration into “high‐risk” and “low‐risk” patients performed by 30% of surveyed centers [30]. It is likely that these practices are maintained because of limited data in this patient population, so we hope our data serve as evidence to support guideline‐based recommendations.

This study also had strengths. The sample size of 192 is larger than any prior study of perioperative antibiotic prophylaxis duration in LTRs and includes a large number of patients with more severe liver failure who were hospitalized prior to transplant [2, 3, 10]. This study confirms and extends the findings of previous studies that excluded severely sick patients [2]. While retrospective, the pre‐post design helps to mitigate selection bias. We included both a specific and a more sensitive definition of post‐operative infection. In addition, even though perioperative prophylaxis is not designed to prevent non‐SSI infections (e.g., pneumonia, urinary tract infection), our sensitivity analysis that captured initiation or escalation of antibiotics for any reason also demonstrated no association between shorter versus longer perioperative antibiotics with suspected infection overall.

In summary, our study found no significant difference in risk of SSI or bacteremia with shorter durations of perioperative antibiotic prophylaxis in LTRs, stratified by risk group, consistent with findings for other surgeries and prior studies in LTRs. Patients admitted prior to transplant did not have increased rates of SSI, may not benefit from a longer duration of perioperative antibiotic prophylaxis than patients with lower MELD scores, and should be included in future studies that examine this question. Further prospective studies are needed to confirm these findings in LTRs; optimizing perioperative antibiotics is an opportunity for antimicrobial stewardship.

## Funding

The authors have nothing to report.

## Conflicts of Interest

The authors declare no conflicts of interest.

## Supporting information




**Table S1**: Demographics by risk group.
**Table S2**: Organisms isolated from blood and SSI cultures within 30 days post‐transplant.
**Table S3**: Outcomes and complications by perioperative antibiotic prophylaxis duration and risk group.
**Table S4**: Univariable and multivariable models of composite definition of infection for sensitivity analysis.
**Table S5**: Univariable and multivariable models of bacteremia and SSI at 100 days post‐transplant sensitivity analysis.
**Table S6**: Outcomes and characteristics by DCD status.
**Figure S1**: Perioperative antibiotic choice.
**Figure S2**: Complications by cohort and risk group.
