# Supplementary material for: No Increased Risk of Infection Following a Protocol Change to Decrease Duration of Perioperative Antibiotic Prophylaxis in Liver Transplantation
Source: Transpl Infect Dis. 2026 Feb 25;28(3):e70192. doi: 10.1111/tid.70192 (PMC13262561; doi:10.1111/tid.70192)
Supplement: Supplementary file 1 — Table S1: Demographics by risk group. Table S2: Organisms isolated from blood and SSI cultures within 30 days post‐transplant. Table S3: Outcomes and complications by perioperative antibiotic prophylaxis duration and risk group. Table S4: Univariable and multivariable models of composite definition of infection for sensitivity analysis. Table S5: Univariable and multivariable models of bacteremia and SSI at 100 days post‐transplant sensitivity analysis. Table S6: Outcomes and characteristics by DCD status. Figure S1: Perioperative antibiotic choice. Figure S2: Complications by cohort and risk group. [file TID-28-e70192-s001.docx]

**Supplementary Tables and Figures**

| Supplemental Table 1. Demographics by risk group. | | | |
| --- | --- | --- | --- |
| **Characteristic** | **Overall**, N = 192 | **Group A**, N = 114 | **Group B**, N = 78 |
| **Age group (years)** |  | | |
| ≥65 | 36 (19%) | 25 (22%) | 11 (14%) |
| 18-44 | 44 (23%) | 19 (17%) | 25 (32%) |
| 45-64 | 112 (58%) | 70 (61%) | 42 (54%) |
| **Male** | 123 (64%) | 73 (64%) | 50 (64%) |
| **Patient defined race** |  | | |
| American Indian and Alaska Native | 7 (3.6%) | 4 (3.5%) | 3 (3.8%) |
| Asian | 2 (1.0%) | 1 (0.9%) | 1 (1.3%) |
| Native Hawaiian and Other Pacific Islander | 1 (0.5%) | 1 (0.9%) | 0 |
| Other | 21 (11%) | 7 (6.1%) | 14 (18%) |
| White or Caucasian | 161 (84%) | 101 (89%) | 60 (77%) |
| **Ethnicity** |  |  |  |
| Choose not to disclose | 2 (1.0%) | 1 (0.9%) | 1 (1.3%) |
| Hispanic/Latino | 28 (15%) | 12 (11%) | 16 (21%) |
| Not Hispanic/Latino | 162 (84%) | 101 (89%) | 61 (78%) |
| **Previous transplant** | 2 (1.0%) | 1 (0.9%) | 1 (1.3%) |
| **MELD score** | 27 (21, 35) | 23 (18, 27) | 36 (33, 40) |
| **Listed 1A** | 5 (2.6%) | 0 | 5 (6.4%) |
| **Surgery duration, minutes, median (Q1, Q3)** | 346 (294, 406) | 346 (289, 406) | 345 (300, 405) |
| **Diabetes** | 85 (44%) | 41 (36%) | 44 (56%) |
| **Beta-lactam allergy** | 27 (14%) | 17 (15%) | 10 (13%) |
| **Cold ischemia time, minutes, median (Q1, Q3)** | 338 (272, 442) | 317 (260, 384) | 390 (303, 468) |
| **Antibiotic exposure within 72 hours prior to transplant**^†^ | 92 (48%) | 32 (28%) | 60 (77%) |
| **SBP prophylaxis prior to transplant** | 22 (11%) | 17 (15%) | 5 (6.4%) |
| **Steroid exposure prior to transplant** | 4 (2.1%) | 2 (1.8%) | 2 (2.6%) |
| **Other immunosuppressants prior to transplant** | 16 (8.3%) | 10 (8.8%) | 6 (7.7%) |
| **CMV serostatus** |  | | |
| D-/R- | 33 (17%) | 21 (18%) | 12 (15%) |
| D-/R+ | 44 (23%) | 27 (24%) | 17 (22%) |
| D+/R- | 41 (21%) | 27 (24%) | 14 (18%) |
| D+/R+ | 74 (39%) | 39 (34%) | 35 (45%) |
| Abbreviations: MELD=Model for End-Stage Liver Disease, Q1=First quartile, Q3=Third quartile, SBP=Spontaneous bacterial peritonitis, D=Donor CMV IgG, R=Recipient CMV IgG | | | |

Supplemental Figure 1. Perioperative antibiotic choice


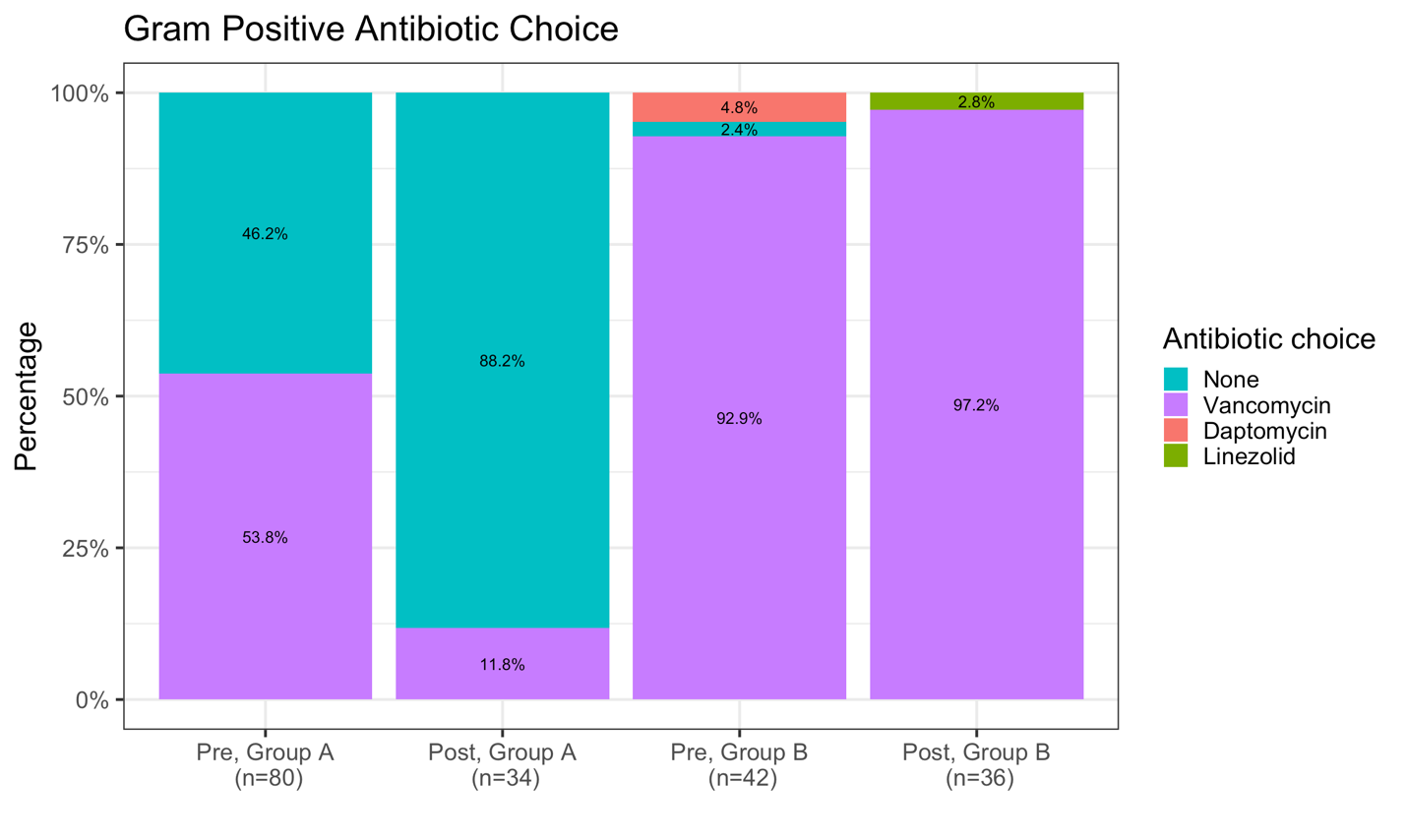


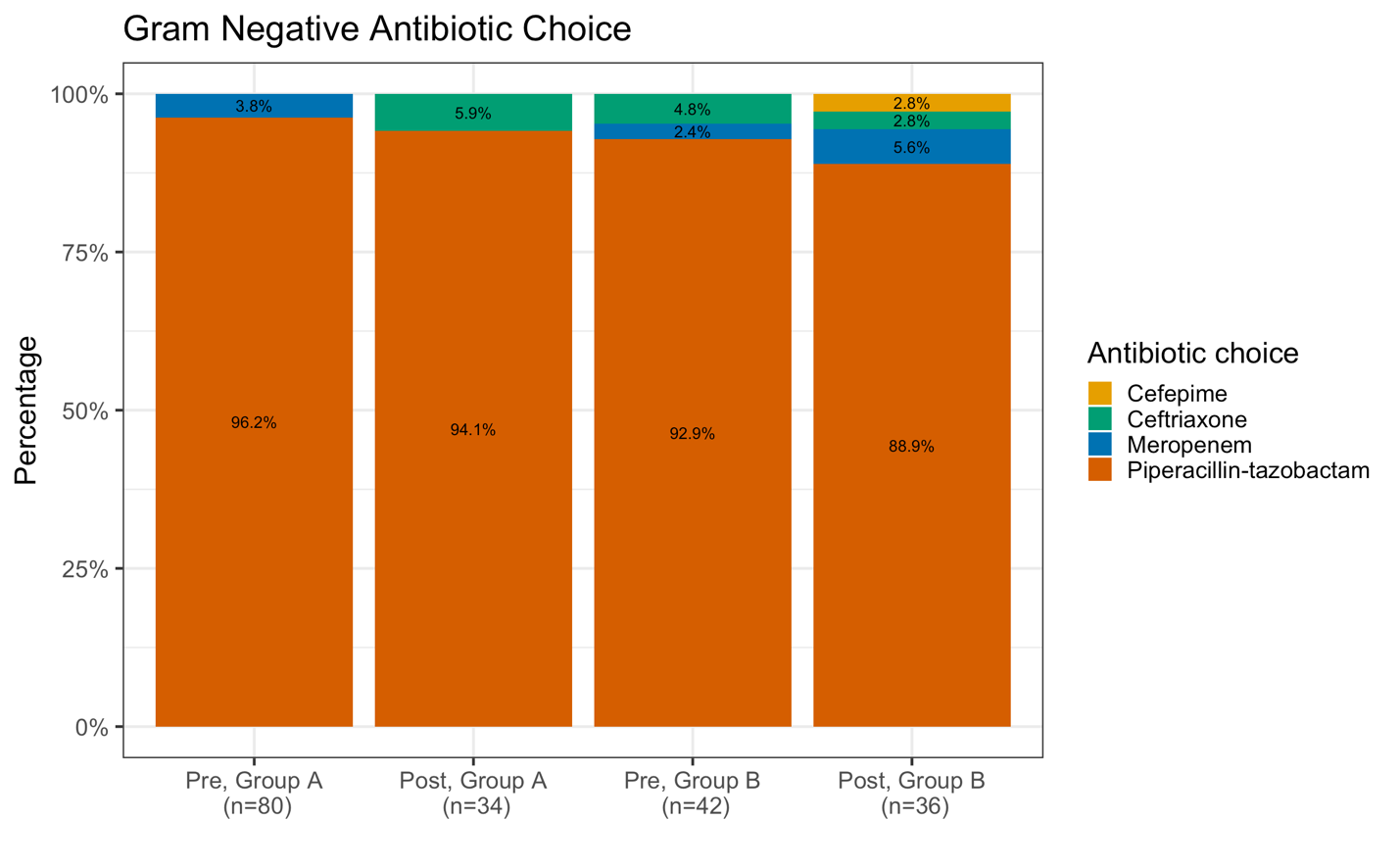


Supplemental Table 2. Organisms isolated from blood and SSI cultures within 30 days post-transplant

| **Organism** | **Time of isolation post-transplant,** (days) |
| --- | --- |
| **Isolated from blood** |  |
| **Group A**, N = 3 |  |
| *Escherichia coli* | 16 |
| *Klebsiella pneumoniae* | 13 |
| *Klebsiella pneumoniae* | 20 |
| **Group B**, N = 0 |  |
| **Isolated from surgical site infection** |  |
| **Group A**, N = 5 |  |
| *Enterobacter cloacae*^†^ | 10 |
| *Enterobacter cloacae*^†^ | 25 |
| *Enterococcus faecium*^†^ | 25 |
| *Enterococcus gallinarum*^†^ | 21 |
| *Klebsiella oxytoca* | 23 |
| *Klebsiella pneumoniae*^†^ | 21 |
| *Pseudomonas aeruginosa* | 10 |
| *Pseudomonas aeruginosa*^†^ | 21 |
| **Group B**, N = 2 |  |
| *Morganella morganii* | 19 |
| *Escherichia coli* | 11 |
| ^†^Multiple organisms were isolated from some specimens | |

Supplemental Table 3. Outcomes and complications by perioperative antibiotic prophylaxis duration and risk group.

|  | **Group A**^†^ |  | **Group B**^†^ |  |
| --- | --- | --- | --- | --- |
|  | **Short (≤72 hours)**, N = 83 (%) | **Long (>72 hours)**, N = 31 (%) | **Short (≤72 hours)**, N = 13 (%) | **Long (>72 hours**, N = 65  (%) |
| **Bacteremia within 30 days** | 2 (2.4) | 1 (3.2) | 0 | 0 |
| **SSI within 30 days** | 2 (2.4) | 3 (9.7) | 1 (7.7) | 1 (1.5) |
| **Bacteremia or SSI within 30 days** | 4 (4.8) | 3 (9.7) | 1 (7.7) | 1 (1.5) |
| **Bacteremia or SSI within 100 days** | 8 (9.6) | 4 (13) | 1 (7.7) | 4 (6.2) |
| **Antibiotic change for suspected infection** | 32 (39) | 13 (42) | 4 (31) | 32 (49) |
| **Infection composite**^‡^ | 32 (39) | 13 (42) | 4 (31) | 32 (49) |
| **Length of stay, days, median (Q1, Q3)** | 8 (6, 11) | 11 (8, 20) | 10 (6, 13) | 19 (11, 26) |
| **Readmission** | 23 (28) | 12 (39) | 1 (7.7) | 12 (18) |
| **30-day mortality** | 0 | 0 | 0 | 0 |
| **AKI**^§^ | 54 (65) | 24 (77) | 11 (85) | 60 (92) |
| **Resistant organism**^¶^ | 7 (8.4) | 7 (23) | 2 (15) | 13 (20) |
| ***Clostridioides difficile* infection** | 8 (9.6) | 3 (9.7) | 1 (7.7) | 6 (9.2) |
| Abbreviations: SSI=Surgical site infection, Q1=First quartile, Q3=Third quartile, AKI=Acute kidney injury  ^†^Group A=Hospitalized <24 hours prior to transplant surgery; Group B=Hospitalized ≥24 hours prior to transplant surgery  ^‡^Bacteremia, SSI or antibiotic change for suspected infection  ^§^Defined by creatinine increase of 0.3 mg/dL in 48 hours, an increase of 1.5x in 7 days, or need for continued renal replacement therapy  ^¶^Methicillin-resistant *Staphylococcus aureus* (n=0), vancomycin-resistant Enterococcus spp. (n=12), enterobacterales with ceftriaxone resistance (n=21), *Pseudomonas* with resistance to ≥3 drug classes to which it is typically susceptible (n=2), *Acinetobacter* spp. (n=0), or *Stenotrophomonas* spp (n=0), patients may have had more than one organism identified. | | | | |

Supplemental Table 4. Univariable and multivariable models of composite definition of infection for sensitivity analysis.

| **Sensitivity analysis for infection**^†^ | | | | |
| --- | --- | --- | --- | --- |
|  | **Univariable** | | **Multivariable** | |
|  | OR (95% CI) | p-value | OR (95% CI) | p-value |
| **Intervention cohort** | 1.14 (0.63, 2.07) ^‡^ | 0.66 | 1.08 (0.59, 1.98) | 0.80 |
| **Group B** | 1.31 (0.73, 2.36) | 0.36 | 1.40 (0.76, 2.56) | 0.28 |
| **Donation after cardiac death** | 2.37 (0.56, 11.8) | 0.25 | 2.73 (0.64, 13.9) | 0.18 |
| **Prophylaxis duration ≥ 72 hours** | 0.68 (0.38, 1.21) | 0.19 |  |  |
| **Age ≥ 65 years** | 1.48 (0.71, 3.07) | 0.29 |  |  |
| **BMI ≥ 30** | 1.48 (0.82, 2.66) | 0.19 |  |  |
| **Diabetes** | 1.31 (0.74, 2.34) | 0.36 |  |  |
| **MELD ≥ 35** | 1.74 (0.93, 3.27) | 0.085 |  |  |
| ^†^Defined as bacteremia, surgical site infection, or antibiotic change for suspected infection  ^‡^An OR < 1 is consistent with a lower risk of that outcome in the post-intervention cohort than the pre-intervention group cohort | | | | |

Supplemental Table 5. Univariable and multivariable models of bacteremia and SSI at 100 days post-transplant sensitivity analysis

| **Bacteremia or SSI at 100 days** | | | | |
| --- | --- | --- | --- | --- |
|  | **Univariable** | | **Multivariable** | |
|  | OR (95% CI) | p-value | OR (95% CI) | p-value |
| **Intervention cohort** | 0.47 (0.13, 1.37) ^†^ | 0.20 | 0.47 (0.13, 1.4) | 0.21 |
| **Group B** | 0.71 (0.24, 1.91) | 0.51 | 0.90 (0.29, 2.57) | 0.84 |
| **Donation after cardiac death** | 3.50 (0.49, 16.7) | 0.14 | 3.44 (0.46, 18.0) | 0.17 |
| **Prophylaxis duration ≥ 72 hours** | 1.00 (0.37, 2.68) | >0.99 |  |  |
| **Age ≥ 65 years** | 0.85 (0.19, 2.78) | 0.81 |  |  |
| **BMI ≥ 30** | 2.7 (1.01, 7.67) | 0.051 |  |  |
| **Diabetes** | 1.29 (0.48, 3.46) | 0.61 |  |  |
| **MELD ≥ 35** | 0.93 (0.29, 2.6) | 0.89 |  |  |
| ^†^An OR < 1 is consistent with a lower risk of that outcome in the post-intervention cohort than the pre-intervention group cohort | | | | |

Supplemental Figure 2. Complications by cohort and risk group.


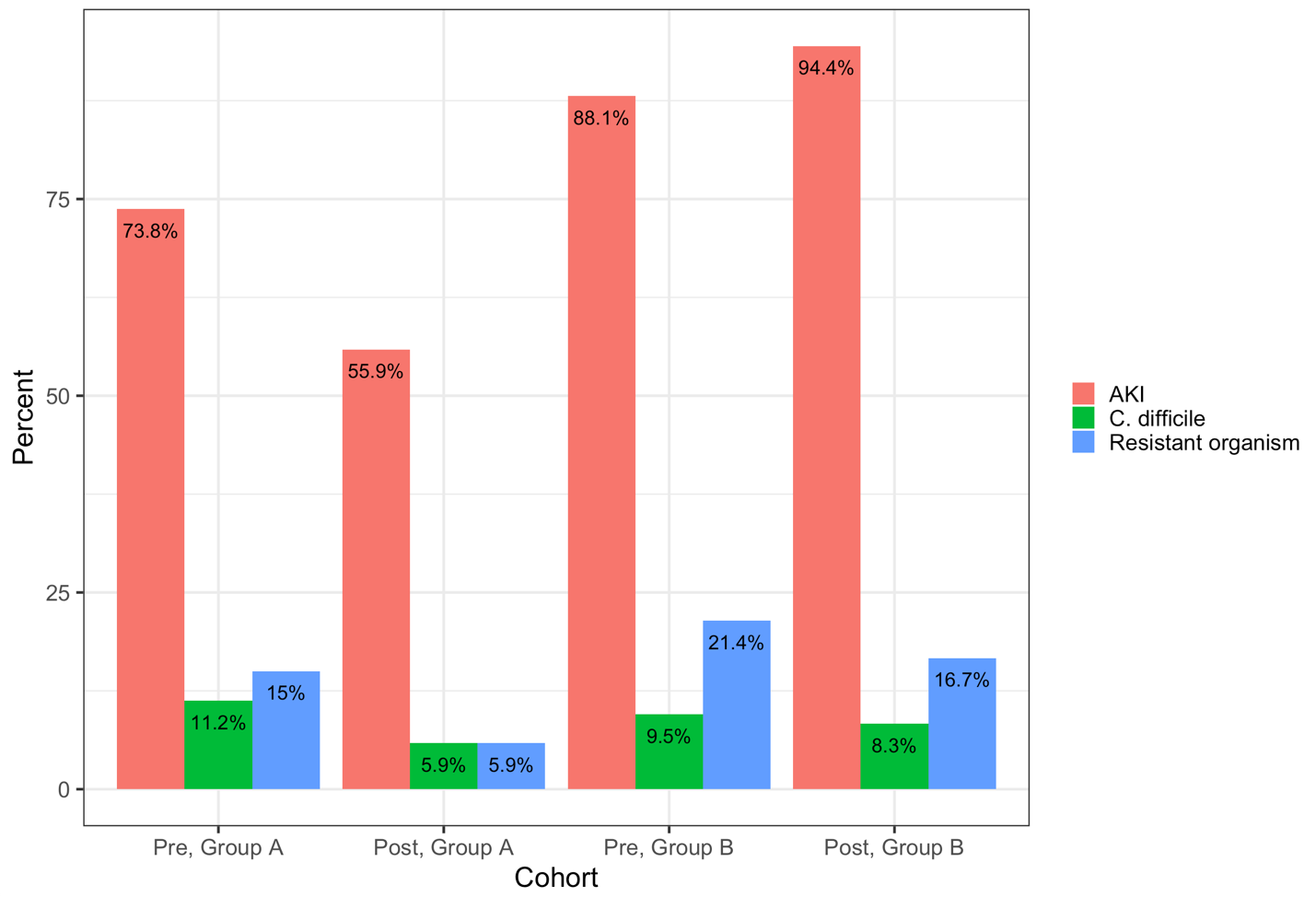


Supplemental Table 6. Outcomes and characteristics by DCD status

|  | **DCD**, N = 8 (%) | **Other**, N = 184 (%) | **p-value** |
| --- | --- | --- | --- |
| **Bacteremia or SSI within 30 days** | 2 (25) | 7 (3.8) | 0.047 |
| **SSI within 30 days** | 2 (25) | 5 (2.7) | 0.029 |
| **Length of stay, days, median (Q1, Q3)** | 10 (6, 17) | 11 (7, 19) | 0.49 |
| **Cold ischemia time, minutes, median (Q1, Q3)** | 344 (310, 374) | 338 (272, 445) | 0.97 |
| **Surgery duration, minutes, median (Q1, Q3)** | 366 (320, 428) | 346 (292, 406) | 0.48 |
| Abbreviations: DCD=Donation after Cardiac Death, SSI=Surgical site infection, Q1=First quartile, Q3=Third quartile | | | |
